# Supplementary material for: 5-Hydroxymethylcytosine signatures in cell-free DNA provide information about tumor types and stages
Source: Cell Res. 2017 Aug 18;27(10):1231–42. doi: 10.1038/cr.2017.106 (PMC5630676; doi:10.1038/cr.2017.106)
Supplement: Supplementary information, Figure S1 — Cell-free 5hmC sequencing by modified hMe-Seal. [file cr2017106x1.pdf]

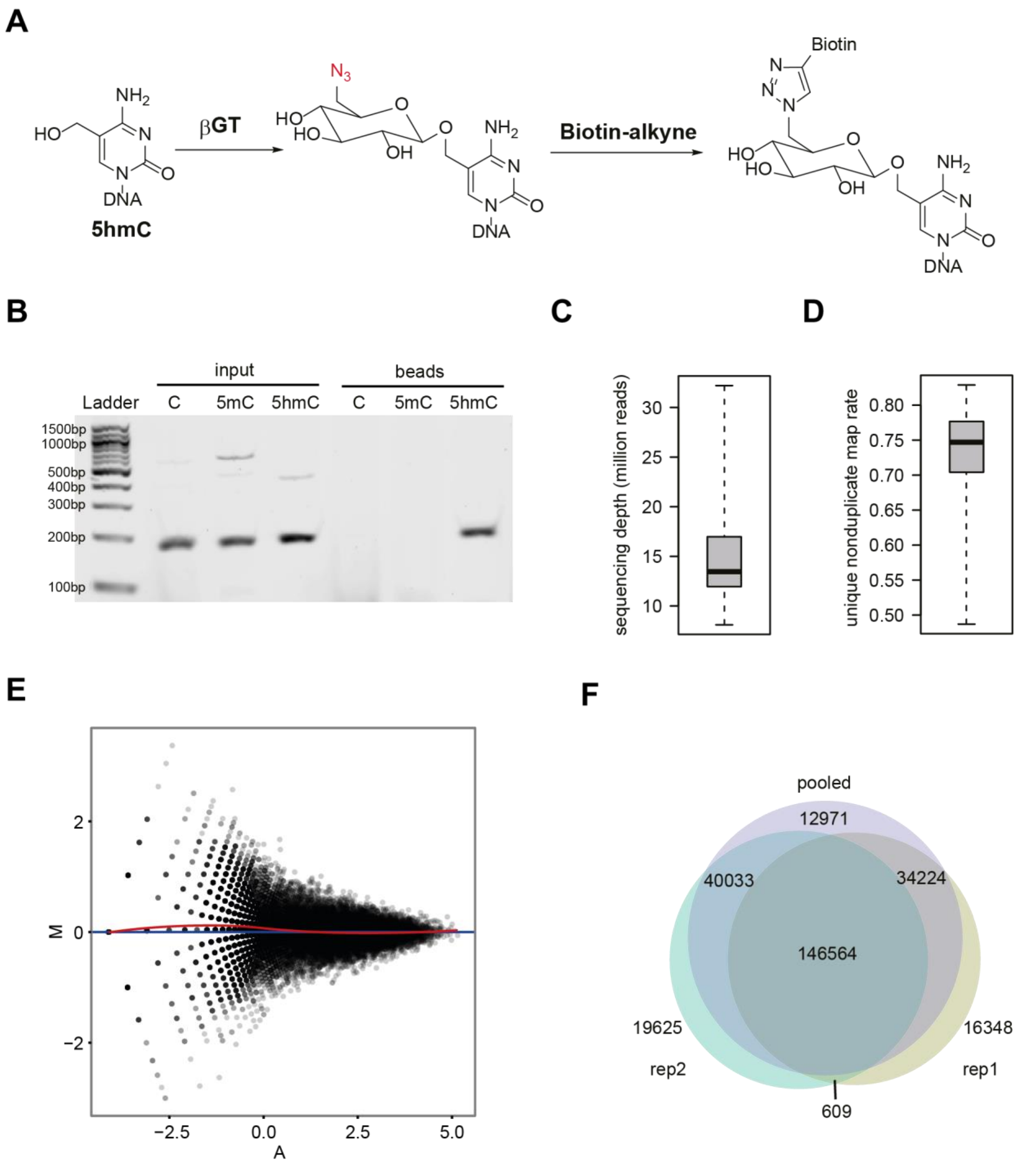

Figure S1 Cell-free 5hmC sequencing by modified hMe-Seal. (**A**) hMe-Seal reactions. 5hmC in DNA is labeled with an azide-modified glucose by βGT, which is then linked to a biotin group through click chemistry. (**B**) Enrichment tests of a single pool of amplicons containing C, 5mC or 5hmC spiked into cfDNA. Showing gel analysis that after hMe-Seal, only 5hmC-containing amplicon can be PCRed from the streptavidin beads. (**C**) Boxplot of sequencing depth across all cell-free samples. (**D**) Boxplot of unique nonduplicate map rate across all cell-free samples. (**E**) MA-plot of normalized cell-free 5hmC read counts (reads/million) in 10 kb bins genome-wide between technical duplicate. The horizontal blue line M = 0 indicates same value in two sample. A lowess fit (in red) is plotted underlying a possible trend in the bias related to the mean value. (**F**) Venn diagram of hMRs overlap between technical replications of cell-free 5hmC sequencing and a pooled sample from both replicates.
