# Supplementary material for: 5-Hydroxymethylcytosine signatures in cell-free DNA provide information about tumor types and stages
Source: Cell Res. 2017 Aug 18;27(10):1231–42. doi: 10.1038/cr.2017.106 (PMC5630676; doi:10.1038/cr.2017.106)
Supplement: Supplementary information, Figure S2 — Genome-wide distribution of 5hmC in cfDNA. [file cr2017106x2.pdf]

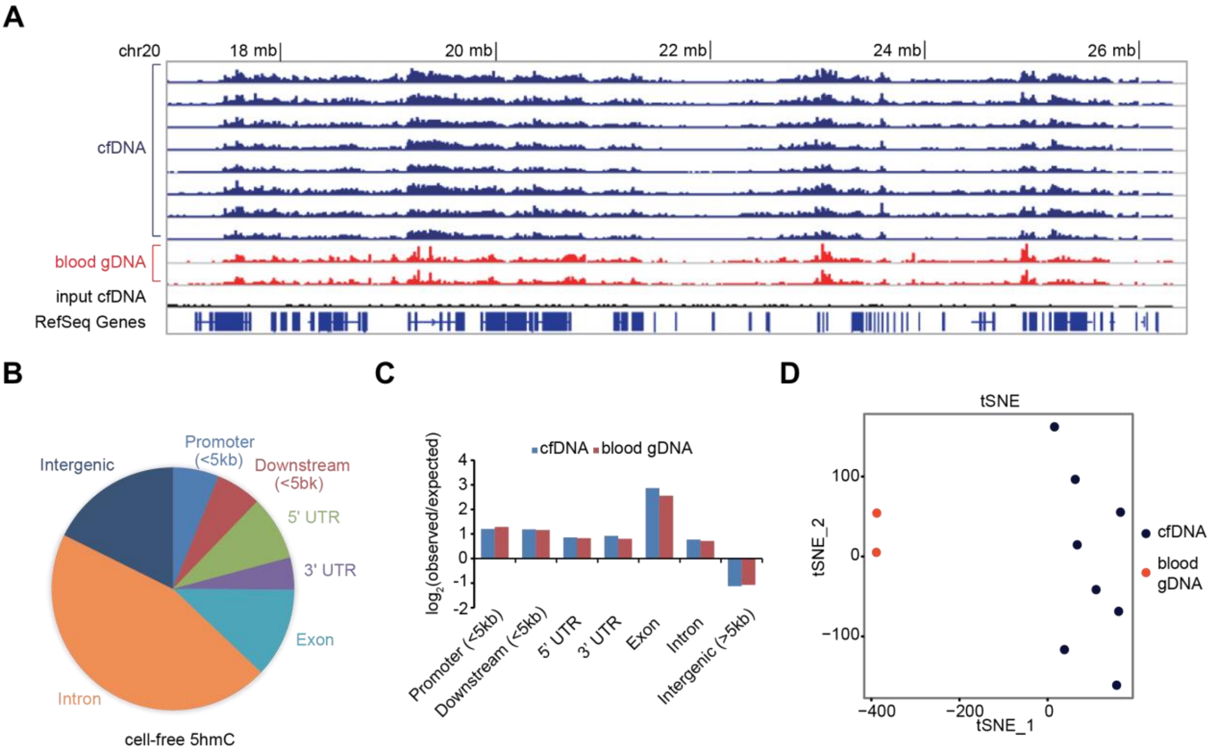

Figure S2 Genome-wide distribution of 5hmC in cfDNA. (**A**) Genome browser view of the 5hmC distribution in a 10 mb region in chromosome 20. Showing the tracks of enriched cfDNA and whole blood gDNA samples along with the unenriched input cfDNA. (**B**) Pie chart presentation of the overall genomic distribution of hMRs in cfDNA. (**C**) The relative enrichment of hMRs across distinct genomic regions in cfDNA and whole blood gDNA. (**D**) tSNE plot of 5hmC FPKM in cfDNA and whole blood gDNA from healthy samples.
