# Supplementary material for: 5-Hydroxymethylcytosine signatures in cell-free DNA provide information about tumor types and stages
Source: Cell Res. 2017 Aug 18;27(10):1231–42. doi: 10.1038/cr.2017.106 (PMC5630676; doi:10.1038/cr.2017.106)
Supplement: Supplementary information, Figure S3 — Differential 5hmC signals between cfDNA and whole blood gDNA. [file cr2017106x3.pdf]

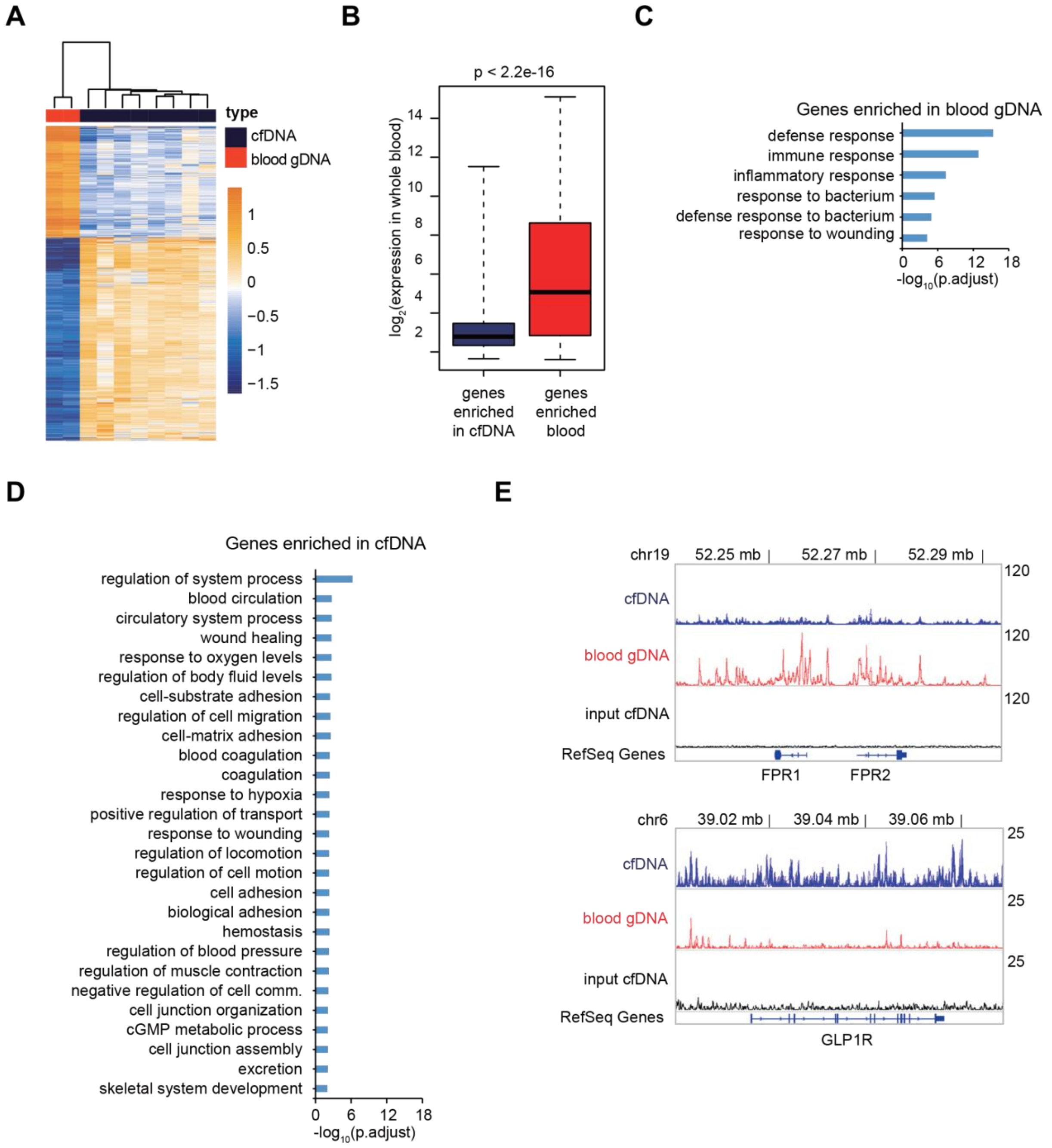

Figure S3 Differential 5hmC signals between cfDNA and whole blood gDNA. (**A**) Heatmap of 2,082 differential genes between cfDNA and blood gDNA. Hierarchical clustering was performed across genes and samples. (**B**) Boxplot of expression level in whole blood for cfDNA and whole blood gDNA 5hmC enriched genes. The *p*-value is shown on top. (**C**, **D**) GO analysis of the whole blood-specific (**C**) and cfDNA-specific (**D**) 5hmC enriched genes, adjusted *p*-value cut off 0.001. (**E**) Genome browser view of the 5hmC distribution in the FPR1/FPR2 (top) and the GLP1R (bottom) loci. Showing the overlapping tracks of cfDNA, whole blood gDNA and input cfDNA in line plot.
