# Supplementary material for: 5-Hydroxymethylcytosine signatures in cell-free DNA provide information about tumor types and stages
Source: Cell Res. 2017 Aug 18;27(10):1231–42. doi: 10.1038/cr.2017.106 (PMC5630676; doi:10.1038/cr.2017.106)
Supplement: Supplementary information, Figure S4 — Cell-free hydroxymethylome in lung cancer. [file cr2017106x4.pdf]

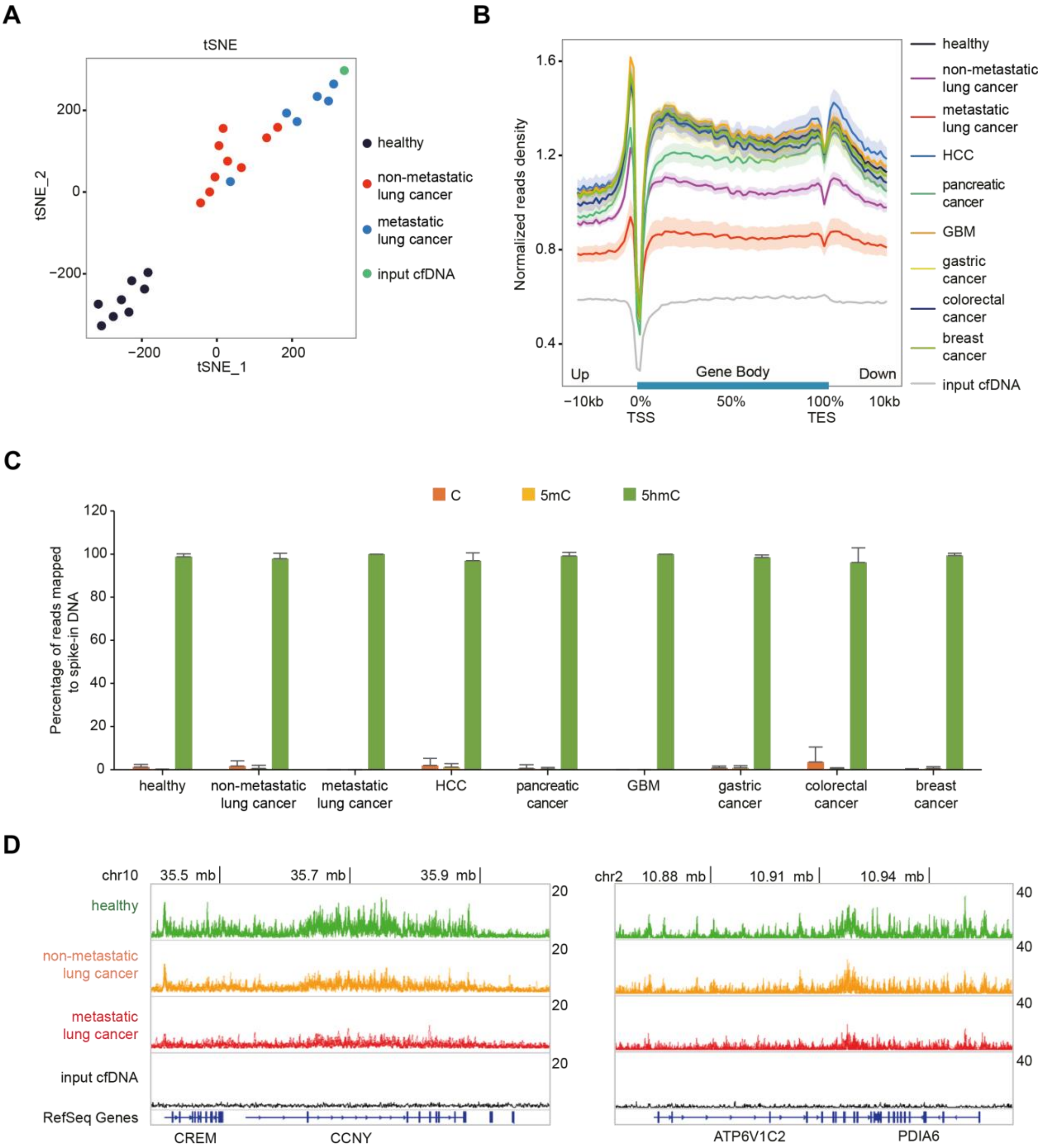

Figure S4 Cell-free hydroxymethylome in lung cancer. (**A**) tSNE plot of 5hmC FPKM from healthy, non-metastatic lung cancer and metastatic lung cancer samples, along with the unenriched input cfDNA. (**B**) Metagene profiles of cell-free 5hmC in healthy and various cancer groups, along with unenriched input cfDNA. Shaded area indicates s.e.m. (**C**) Percentage of reads mapped to spike-in DNA in the sequencing libraries of various groups. Error bars indicate s.d. (**D**) Genome browser view of the cell-free 5hmC distribution in the CREM/CCNY (left) and ATP6V1C2/PDIA6 (right) loci in healthy and lung cancer samples. Showing the overlapping tracks in line plot.
