# Supplementary material for: 5-Hydroxymethylcytosine signatures in cell-free DNA provide information about tumor types and stages
Source: Cell Res. 2017 Aug 18;27(10):1231–42. doi: 10.1038/cr.2017.106 (PMC5630676; doi:10.1038/cr.2017.106)
Supplement: Supplementary information, Figure S5 — Cell-free hydroxymethylome in HCC. [file cr2017106x5.pdf]

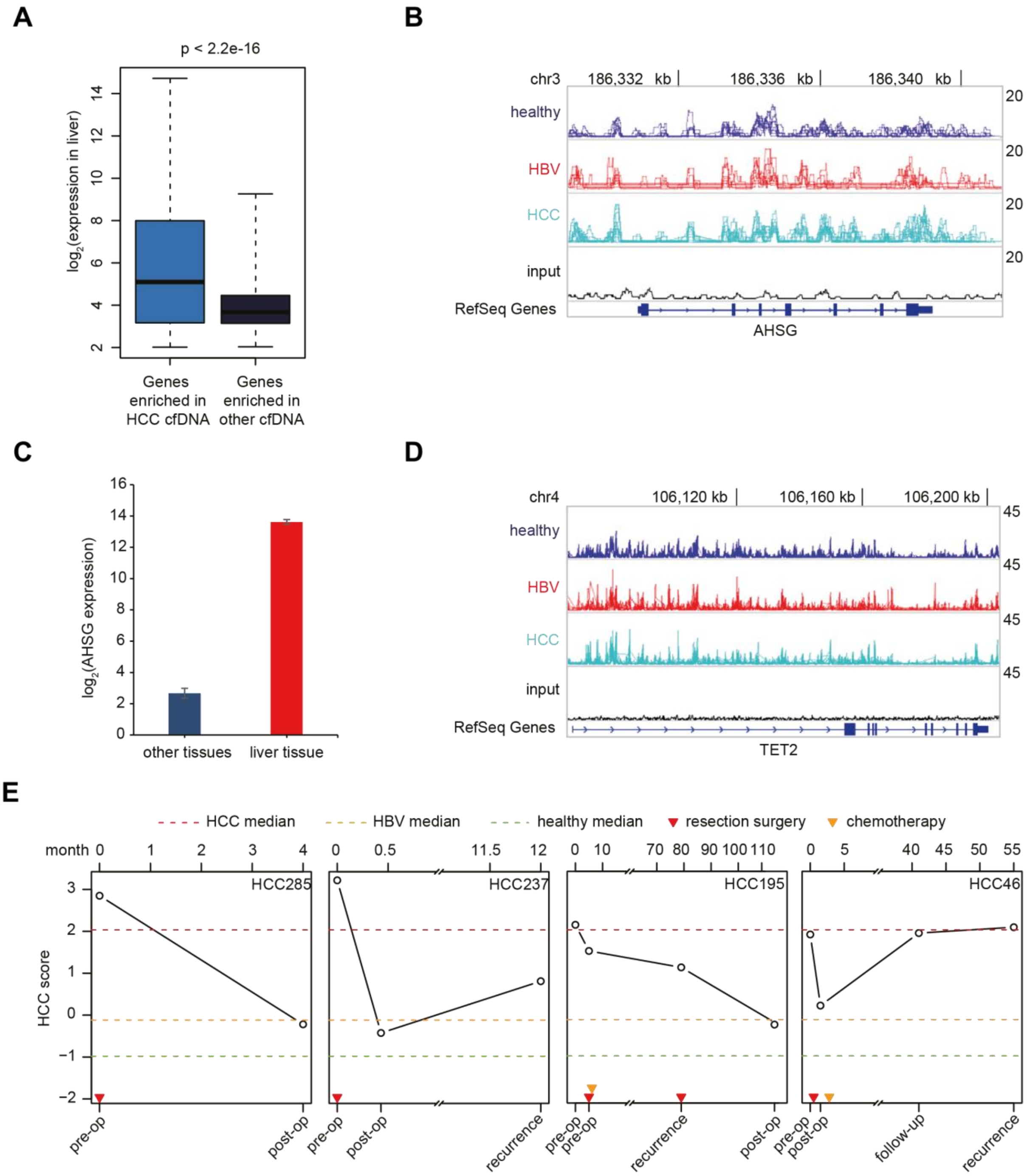

Figure S5 Cell-free hydroxymethylome in HCC. (**A**) Boxplot of expression level in liver tissue for HCC-specific 5hmC enriched and depleted genes. The *p*-value is shown on top. (**B**) Genome browser view of the cell-free 5hmC distribution in the AHSG locus in healthy HBV and HCC samples. Showing the overlapping tracks in line plot. (**C**) Expression of AHSG in liver and other tissues. (**D**) Genome browser view of the cell-free 5hmC distribution in the TET2 locus in healthy, HBV and HCC samples. Showing the overlapping tracks in line plot. (**E**) Changes of HCC score in 4 HCC follow-up cases. Disease status shown on the bottom. Time duration in month shown on the top. Dotted lines indicate the median values of HCC scores in the HCC, HBV, and healthy groups. Triangles indicate treatment. HCC score is a linear combination of 1,006 HCC differential genes (Figure 3B) that best separates HCC from HBV and healthy samples.
