# Supplementary material for: 5-Hydroxymethylcytosine signatures in cell-free DNA provide information about tumor types and stages
Source: Cell Res. 2017 Aug 18;27(10):1231–42. doi: 10.1038/cr.2017.106 (PMC5630676; doi:10.1038/cr.2017.106)
Supplement: Supplementary information, Figure S6 — Cell-free hydroxymethylome in pancreatic cancer. [file cr2017106x6.pdf]

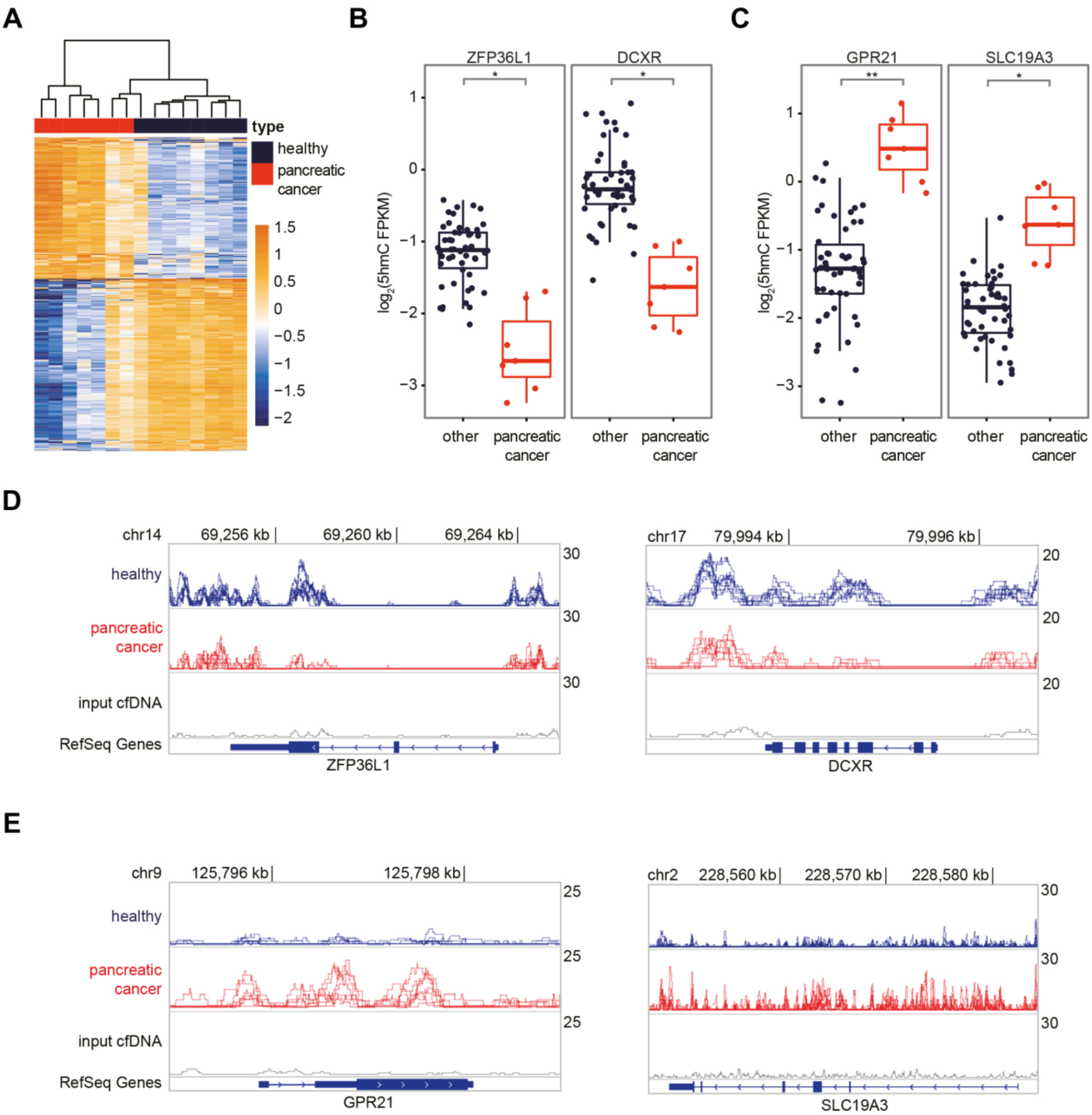

Figure S6 Cell-free hydroxymethylome in pancreatic cancer. (**A**) Heatmap of 713 pancreatic cancer differential genes in healthy and pancreatic cancer samples. Hierarchical clustering was performed across genes and samples. (**B, C**) Boxplots of ZFP36L1, DCXR (**B**) and GPR21, SLC19A3 (**C**) 5hmC FPKM in pancreatic cancer and other cfDNA samples. **P* < 0.001, ***P* <1e-5, Welch t-test. (**D**, **E**) Genome browser view of the cell-free 5hmC distribution in the ZFP36L1, DCXR (**D**) and GPR21, SLC19A3 (**E**) loci in healthy and pancreatic cancer samples. Showing the overlapping tracks in line plot.
