# Supplementary material for: 5-Hydroxymethylcytosine signatures in cell-free DNA provide information about tumor types and stages
Source: Cell Res. 2017 Aug 18;27(10):1231–42. doi: 10.1038/cr.2017.106 (PMC5630676; doi:10.1038/cr.2017.106)
Supplement: Supplementary information, Figure S7 — CNV estimation from input cfDNA and 5hmC enrichment sequencing. [file cr2017106x7.pdf]

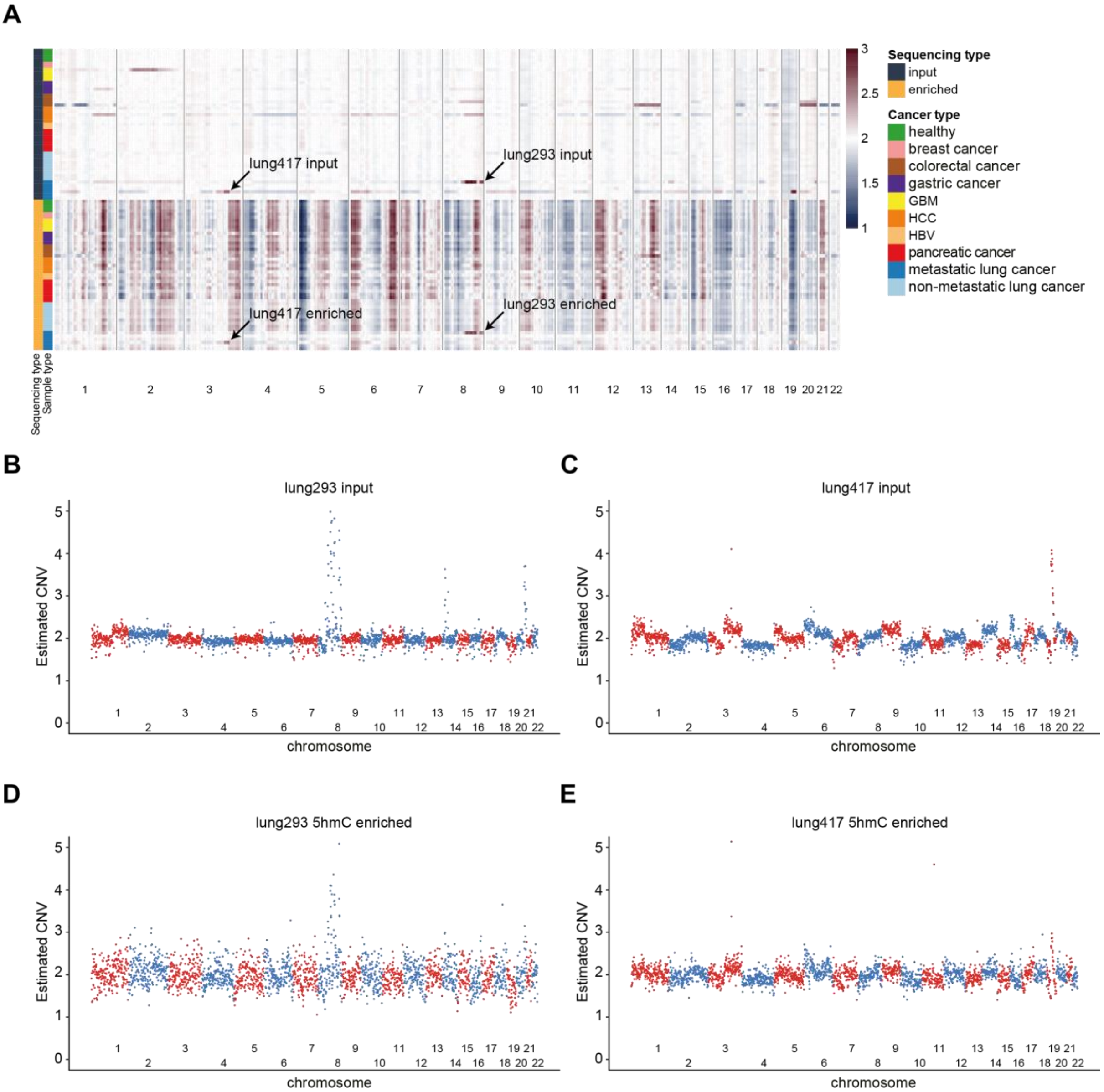

Figure S7 CNV estimation from input cfDNA and 5hmC enrichment sequencing. (**A**) CNV estimation heatmap from input cfDNA and 5hmC enrichment sequencing in 1 mb bin. Averaged bin counts within a sliding window of 20 bins were calculated as the estimated CNV for each bin. No clustering was performed. Arrows indicate samples with matched patterns in input cfDNA and 5hmC enrichment sequencing. (**B, C**) CNV estimation from input cfDNA sequencing of metastatic lung cancer patients lung293 (**B**) and lung417 (**C**). (**D**, **E**) CNV estimation from 5hmC enrichment sequencing of metastatic lung cancer patients lung293 (**D**) and lung417 (**E**).
