# Supplementary material for: 5-Hydroxymethylcytosine signatures in cell-free DNA provide information about tumor types and stages
Source: Cell Res. 2017 Aug 18;27(10):1231–42. doi: 10.1038/cr.2017.106 (PMC5630676; doi:10.1038/cr.2017.106)
Supplement: Supplementary information, Figure S8 — Cell-free hydroxymethylome in cancer samples. [file cr2017106x8.pdf]

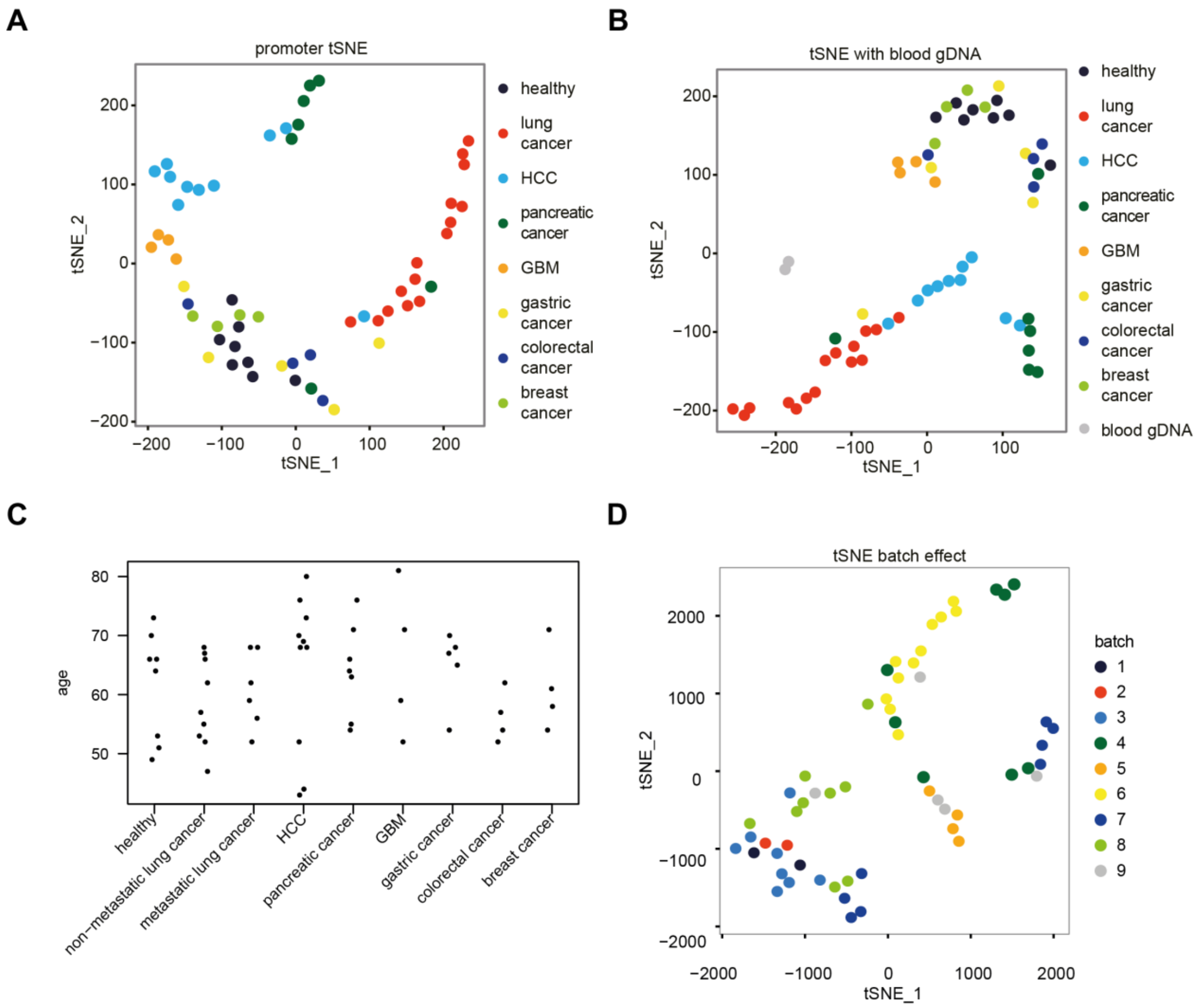

Figure S8 Cell-free hydroxymethylome in cancer samples. (**A**) tSNE plot of 5hmC FPKM in promoter regions (5 kb upstream of TSS) from healthy and various caner samples. (**B**) tSNE plot of 5hmC FPKM from healthy and various caner cfDNA samples along with the whole blood gDNA samples. (**C**) Age distribution of healthy individual and various cancer patients. (**D**) tSNE plot of 5hmC FPKM in cfDNA from healthy and various cancer samples (Figure 4A) colored by batches numbered according to the process time.
