# Supplementary material for: 5-Hydroxymethylcytosine signatures in cell-free DNA provide information about tumor types and stages
Source: Cell Res. 2017 Aug 18;27(10):1231–42. doi: 10.1038/cr.2017.106 (PMC5630676; doi:10.1038/cr.2017.106)
Supplement: Supplementary information, Figure S9 — Cancer type and stage prediction with cell-free 5hmC. [file cr2017106x9.pdf]

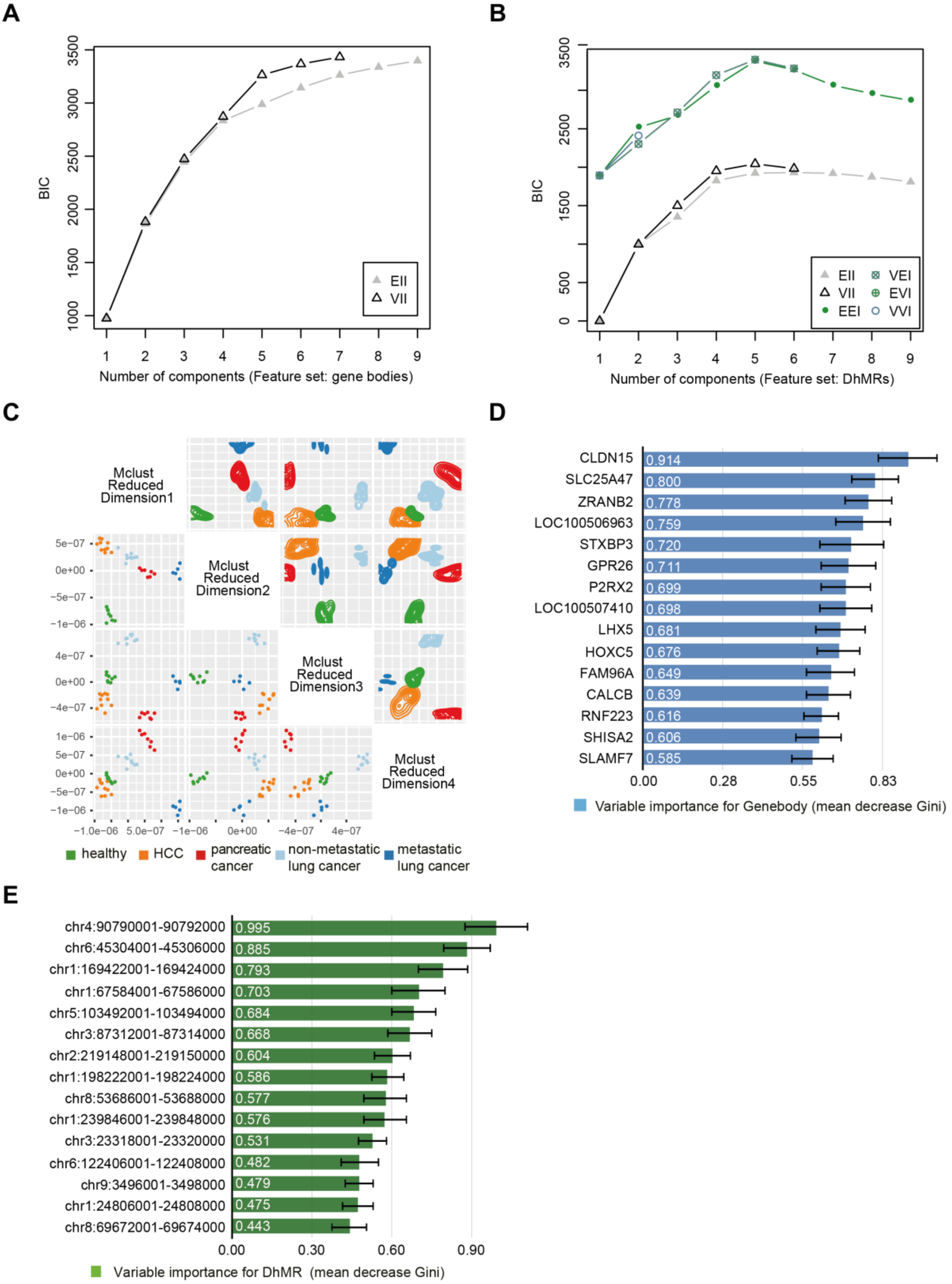

Figure S9 Cancer type and stage prediction with cell-free 5hmC. (**A**, **B**) Bayesian Information Criterion (BIC) plot by Mclust trained with 66 gene body feature set (**A**) and 71 DhMRs feature set from samples other than lung324 (for leave-one-out cross-validation) (**B**), indicating high BIC value for separating five groups when using VII model for gene bodies and EEI for DhMRs. (**C**) 4-Dimensional Mclust-based dimensionality reduction

plot using 66 gene body features from samples other than lung324 (for leave-one-out cross-validation). The lower half shows the scatter plot and the upper half shows the density plot. (**D**, **E**), Variable importance (mean decrease Gini) for the top 15 gene bodies (**D**) and DhMRs (**E**), in the random forest training model. The error bar indicates the standard deviation of variance importance from leave-one-out cross-validation.
