# Supplementary material for: 5-Hydroxymethylcytosine signatures in cell-free DNA provide information about tumor types and stages
Source: Cell Res. 2017 Aug 18;27(10):1231–42. doi: 10.1038/cr.2017.106 (PMC5630676; doi:10.1038/cr.2017.106)
Supplement: Supplementary information, Figure S10 — Examples of DhMRs in the random forest model. [file cr2017106x10.pdf]

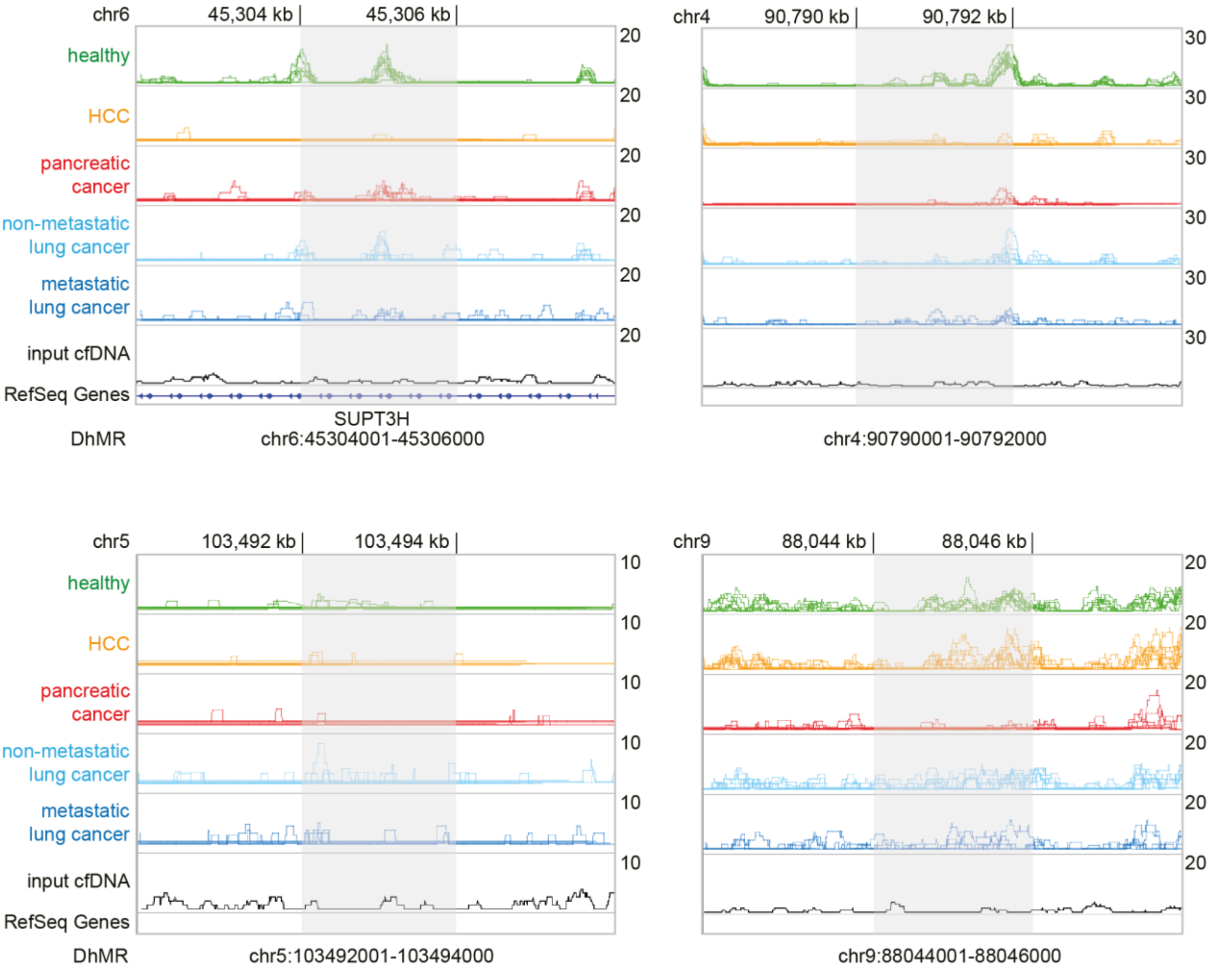

Figure S10 Examples of DhMRs in the random forest model. Genome browser view of the cell-free 5hmC distribution in four DhMRs with high variable importance in the random forest model (Figure S9E) in various groups. Showing the overlapping tracks in line plot. Shaded area indicates the DhMR.
