# Supplementary material for: 5-Hydroxymethylcytosine signatures in cell-free DNA provide information about tumor types and stages
Source: Cell Res. 2017 Aug 18;27(10):1231–42. doi: 10.1038/cr.2017.106 (PMC5630676; doi:10.1038/cr.2017.106)
Supplement: Supplementary information, Table S1 — Summary of 5hmC sequencing results. [file cr2017106x11.pdf]

Table S1 Summary of 5hmC sequencing results.

sample ID	type	total reads sequenced	unique nonduplicate mapped reads	unique nonduplicate mapped rate
**10**	healthy cfDNA	20081973	15192613	0.76
**11**	healthy cfDNA	19142986	14762956	0.77
**27**	healthy cfDNA	21862078	16645192	0.76
**35-1 ***	healthy cfDNA	29132339	16742468	0.57
**35-2 ***	healthy cfDNA	28694218	17346511	0.60
**36-1 ***	healthy cfDNA	32202519	20996955	0.65
**36-2 ***	healthy cfDNA	31089686	20993595	0.68
**38o**	healthy cfDNA	20124203	15295376	0.76
**38**	healthy cfDNA	20419287	15679281	0.77
**39o**	healthy cfDNA	22320662	17833176	0.80
**input §**	cfDNA input	38574253	25910419	0.67
**35-blood**	whole blood gDNA	44077590	31654982	0.72
**36-blood**	whole blood gDNA	40843066	29266169	0.72
**blood-input †**	whole blood gDNA input	39138506	26455609	0.68
**lung293**	lung cancer	14172402	11470840	0.81
**lung323**	lung cancer	12269885	8916594	0.73
**lung324**	lung cancer	13313728	10058078	0.76
**lung395**	lung cancer	13589263	10092883	0.74
**lung417**	lung cancer	13212811	10109574	0.77
**lung418**	lung cancer	13103903	10420656	0.80
**lung419**	lung cancer	11949356	9704240	0.81
**lung492**	lung cancer	12563742	8885504	0.71
**lung493**	lung cancer	12930120	10479700	0.81
**lung496**	lung cancer	12267496	9657956	0.79
**lung512**	lung cancer	12934833	10483836	0.81
**lung513**	lung cancer	11310088	8304508	0.73
**lung514**	lung cancer	12895079	10264145	0.80
**lung515**	lung cancer	12132995	9406700	0.78
**lung517**	lung cancer	11766082	8857054	0.75
**HCC150**	HCC	15215190	11298385	0.74
**HCC237**	HCC	13439935	10109197	0.75
**HCC241**	HCC	16201676	12017320	0.74
**HCC256**	HCC	14579945	10728759	0.74
**HCC260**	HCC	13791503	10021911	0.73
**HCC285**	HCC	11522024	7662330	0.67
**HCC290**	HCC	13162465	9271065	0.70
**HCC320**	HCC	13462633	9696240	0.72
**HCC341**	HCC	11199473	6497400	0.58
**HCC628**	HCC	15365745	11759122	0.77
**HCC324**	HCC	12525818	9598812	0.77

**HCC46**	HCC	13121530	9237102	0.70
**HCC73**	HCC	13816686	10745247	0.78
**HCC398**	HCC	13791599	10430016	0.76
**HCC489**	HCC	11446887	5575387	0.49
**HCC195**	HCC	11538777	7701351	0.67
**HCC234**	HCC	11960087	8468478	0.71
**HCC626**	HCC	13552712	11087605	0.82
**HCC647**	HCC	12491614	8590321	0.69
**pancreatic27**	pancreatic cancer	9717087	8019436	0.83
**pancreatic68**	pancreatic cancer	10457109	8374219	0.80
**pancreatic69**	pancreatic cancer	10838005	8940883	0.82
**pancreatic75**	pancreatic cancer	10197772	8452749	0.83
**pancreatic9**	pancreatic cancer	14601356	11245279	0.77
**pancreatic15**	pancreatic cancer	15240467	11923009	0.78
**pancreatic22**	pancreatic cancer	13439343	10356395	0.77
**GBM57**	GBM	8799132	6455359	0.73
**GBM58**	GBM	8874810	7253089	0.82
**GBM66**	GBM	9795211	8073651	0.82
**GBM76**	GBM	8103209	6165341	0.76
**stomach1**	gastric cancer	14282633	10365849	0.73
**stomach2**	gastric cancer	17825012	12938872	0.73
**stomach3**	gastric cancer	16979690	12894400	0.76
**stomach4**	gastric cancer	21192604	15675499	0.74
**stomach8**	gastric cancer	14070772	8321549	0.59
**colon13**	colorectal cancer	17352371	12517451	0.72
**colon16**	colorectal cancer	15470656	11210513	0.72
**colon17**	colorectal cancer	15101557	10590748	0.70
**colon19**	colorectal cancer	18441208	12503926	0.68
**BR5-1 ***	breast cancer	17826666	13542700	0.76
**BR5-2 ***	breast cancer	17746176	13004851	0.73
**BR7-1 ***	breast cancer	16963664	13160842	0.78
**BR7-2 ***	breast cancer	15495003	12100951	0.78
**BR13**	breast cancer	21382473	16015986	0.75
**BR14**	breast cancer	18668112	14613260	0.78
**HBV268**	HBV	8730571	5106519	0.58
**HBV334**	HBV	11838111	7848078	0.66
**HBV374**	HBV	14896634	11099981	0.75
**HBV397**	HBV	12127855	8416798	0.69
**HBV455**	HBV	12796382	9001735	0.70
**HBV640**	HBV	10040349	6062886	0.60
**HBV646**	HBV	9665264	5002160	0.52

* Technical duplicate.

§ Unenriched input cfDNA, mixed from samples 35 and 36.

† Unenriched input whole blood gDNA, mixed from samples 35-blood and 36-blood.
