# Supplementary material for: 5-Hydroxymethylcytosine signatures in cell-free DNA provide information about tumor types and stages
Source: Cell Res. 2017 Aug 18;27(10):1231–42. doi: 10.1038/cr.2017.106 (PMC5630676; doi:10.1038/cr.2017.106)
Supplement: Supplementary information, Table S2 — Clinical information for healthy samples. [file cr2017106x12.pdf]

Table S2 Clinical information for healthy samples.

sample ID	gender	age
**10**	female	53
**11**	female	66
**27**	female	66
**35**	male	51
**36**	male	73
**38o**	female	70
**38**	female	64
**39o**	female	49
