# Supplementary material for: 5-Hydroxymethylcytosine signatures in cell-free DNA provide information about tumor types and stages
Source: Cell Res. 2017 Aug 18;27(10):1231–42. doi: 10.1038/cr.2017.106 (PMC5630676; doi:10.1038/cr.2017.106)
Supplement: Supplementary information, Table S3 — Clinical information for lung cancer samples. [file cr2017106x13.pdf]

Table S3 Clinical information for lung cancer samples.

sample ID	category	TNM	stage	gender	age
**lung395**	non-metastatic lung cancer	T4N2Mx	III	female	62
**lung419**	non-metastatic lung cancer	T1N2M0G2	IIIa	female	53
**lung492**	non-metastatic lung cancer	T2N0M0	I	male	55
**lung493**	non-metastatic lung cancer	T1N3M0	IV	female	66
**lung496**	non-metastatic lung cancer	T3N1M0	IIIa	male	68
**lung512**	non-metastatic lung cancer	-	-	female	67
**lung513**	non-metastatic lung cancer	T2N1M0	I-II	male	47
**lung514**	non-metastatic lung cancer	T2N0M0	I-II	female	57
**lung515**	non-metastatic lung cancer	cT3N1M0	IIIA	male	52
**lung293**	metastatic lung cancer	cT4N3M1a	IV	female	52
**lung323**	metastatic lung cancer	TxN2M1	IV	female	68
**lung324**	metastatic lung cancer	TxNxM1	IV	male	56
**lung417 §**	metastatic lung cancer	-	-	male	62
**lung418**	metastatic lung cancer	TxN3Mx	IIIb-IV	male	59
**lung517**	metastatic lung cancer	cT4N2M1b	IV	male	68

All are non-small cell lung cancer samples unless otherwise noted.

§ Small cell lung cancer.
