# Supplementary material for: 5-Hydroxymethylcytosine signatures in cell-free DNA provide information about tumor types and stages
Source: Cell Res. 2017 Aug 18;27(10):1231–42. doi: 10.1038/cr.2017.106 (PMC5630676; doi:10.1038/cr.2017.106)
Supplement: Supplementary information, Table S4 — Clinical information for HCC samples. [file cr2017106x14.pdf]

Table S4 Clinical information for HCC samples.

sample ID	category	TNM	tumor size (cm)	gender	age
**HBV268**	HBV	-	-	male	36
**HBV334**	HBV	-	-	female	55
**HBV374**	HBV	-	-	female	45
**HBV397**	HBV	-	-	female	51
**HBV455**	HBV	-	-	female	66
**HBV640**	HBV	-	-	female	49
**HBV646**	HBV	-	-	male	60
**HCC150**	HCC pre-op	pT1 pNX pMX	3.1 §	male	76
**HCC256**	HCC pre-op	pT1 pNX pMX	15x9	male	80
**HCC260**	HCC pre-op	pT1 pNX pMX	1.3 §	male	68
**HCC290**	HCC pre-op	-	10x13x18	male	68
**HCC320**	HCC pre-op	-	multifocal	female	70
**HCC628**	HCC pre-op	pT1	1.8 §	male	43
**HCC285**	HCC pre-op	pT3N0M0	8 §	male	73
**HCC324**	HCC post-op	-	-		73
**HCC237**	HCC pre-op	pT2 pNX pMX	4.1 §	male	52
**HCC241**	HCC post-op	-	-		52
**HCC341**	HCC recurrence	-	3x1.2		53
**HCC195**	HCC pre-op	pT1 pNX pM0	-	male	44
**HCC234**	HCC pre-op	-	1.6 §		44
**HCC626**	HCC recurrence	pT1 pNX pM0	1.7x1.7x1.0		50
**HCC647**	HCC post-op	-	-		53
**HCC46**	HCC pre-op	pT2 pNX pMX	2.8 §	male	69
**HCC73**	HCC post-op	-	-		69
**HCC398**	HCC follow-up	-	-		72
**HCC489**	HCC recurrence	-	2.2 §		73

Same color shade indicate follow-up of the same patient.

§ in greatest dimension.
