# Supplementary material for: 5-Hydroxymethylcytosine signatures in cell-free DNA provide information about tumor types and stages
Source: Cell Res. 2017 Aug 18;27(10):1231–42. doi: 10.1038/cr.2017.106 (PMC5630676; doi:10.1038/cr.2017.106)
Supplement: Supplementary information, Table S5 — Clinical information for pancreatic cancer samples. [file cr2017106x15.pdf]

Table S5 Clinical information for pancreatic cancer samples.

sample ID	TNM	stage	metastasis to	gender	age
**pancreatic9**	T3N0M1	IV	liver	male	76
**pancreatic15**	T1N0M0	IA	-	male	64
**pancreatic22**	T4N1M0	III	-	female	71
**pancreatic27**	T4N1M1	IV	abdominal wall, omentum	male	55
**pancreatic68**	T3N0M1	IV	liver	male	63
**pancreatic69**	T3N0M0	IIA	-	male	66
**pancreatic75**	T3N0M0	IIA	-	male	54
