# Supplementary material for: 5-Hydroxymethylcytosine signatures in cell-free DNA provide information about tumor types and stages
Source: Cell Res. 2017 Aug 18;27(10):1231–42. doi: 10.1038/cr.2017.106 (PMC5630676; doi:10.1038/cr.2017.106)
Supplement: Supplementary information, Table S6 — Clinical information for GBM samples. [file cr2017106x16.pdf]

**Table S6** Clinical information for GBM samples.

| **sample ID** | **stage** | **gender** | **age** |
|---|---|---|---|
| **GBM57** | IV | female | 52 |
| **GBM58** | IV | male | 71 |
| **GBM66** | IV | male | 81 |
| **GBM76** | IV | male | 59 |
