# Supplementary material for: 5-Hydroxymethylcytosine signatures in cell-free DNA provide information about tumor types and stages
Source: Cell Res. 2017 Aug 18;27(10):1231–42. doi: 10.1038/cr.2017.106 (PMC5630676; doi:10.1038/cr.2017.106)
Supplement: Supplementary information, Table S7 — Clinical information for gastric cancer samples. [file cr2017106x17.pdf]

Table S7 Clinical information for gastric cancer samples.

**sample ID**	**TNM**	**stage**	**gender**	**age**
**stomach1**	T2N1M0	II a	male	67
**stomach2**	T4aN3bM0	III c	male	54
**stomach3**	T1aN0M0	I a	male	68
**stomach4**	T4bN0M0	III b	male	70
**stomach8**	T1bN0M0	I a	male	65
