# Supplementary material for: 5-Hydroxymethylcytosine signatures in cell-free DNA provide information about tumor types and stages
Source: Cell Res. 2017 Aug 18;27(10):1231–42. doi: 10.1038/cr.2017.106 (PMC5630676; doi:10.1038/cr.2017.106)
Supplement: Supplementary information, Table S8 — Clinical information for colorectal cancer samples. [file cr2017106x18.pdf]

Table S8 Clinical information for colorectal cancer samples.

sample ID	TNM	stage	gender	age
**colon13**	T4N0M0	II	male	54
**colon16**	T3N0M0	II	female	57
**colon17**	T4N0M1	IV	male	52
**colon19**	pT4N1M1	IV	female	62
