# Supplementary material for: 5-Hydroxymethylcytosine signatures in cell-free DNA provide information about tumor types and stages
Source: Cell Res. 2017 Aug 18;27(10):1231–42. doi: 10.1038/cr.2017.106 (PMC5630676; doi:10.1038/cr.2017.106)
Supplement: Supplementary information, Table S9 — Clinical information for breast cancer samples. [file cr2017106x19.pdf]

Table S9 Clinical information for breast cancer samples.

sample ID	tumor size (cm)	tumor grade	age
**BR5**	2.5	2	54
**BR7**	1.2	1	71
**BR13**	1	2	58
**BR14**	1.9	1	61
