# Supplementary material for: 5-Hydroxymethylcytosine signatures in cell-free DNA provide information about tumor types and stages
Source: Cell Res. 2017 Aug 18;27(10):1231–42. doi: 10.1038/cr.2017.106 (PMC5630676; doi:10.1038/cr.2017.106)
Supplement: Supplementary information, Table S10 — Summary of input cfDNA sequencing results. [file cr2017106x20.pdf]

Table S10 Summary of input cfDNA sequencing results.

sample ID	type	total reads sequenced	unique nonduplicate mapped reads	unique nonduplicate mapped rate
**10-input**	healthy	12297454	9023854	0.73
**27-input**	healthy	14185485	10553292	0.74
**38o-input**	healthy	12534132	9265385	0.74
**38-input**	healthy	12329856	9068759	0.74
**lung293-input**	lung cancer	14387649	10706785	0.74
**lung323-input**	lung cancer	12718973	9096119	0.72
**lung324-input**	lung cancer	14283775	10338094	0.72
**lung395-input**	lung cancer	12340896	8984866	0.73
**lung417-input**	lung cancer	17296343	12782946	0.74
**lung418-input**	lung cancer	20112071	13065287	0.65
**lung419-input**	lung cancer	17101222	12919260	0.76
**lung492-input**	lung cancer	13523477	8003249	0.59
**lung493-input**	lung cancer	14517791	10169937	0.70
**lung496-input**	lung cancer	11334027	8241433	0.73
**lung512-input**	lung cancer	14193573	10244580	0.72
**lung513-input**	lung cancer	17138456	10633764	0.62
**lung514-input**	lung cancer	14089241	9448179	0.67
**lung515-input**	lung cancer	12218453	8370255	0.69
**lung517-input**	lung cancer	123012581	74444363	0.61
**HCC237-input**	HCC	16868568	11576052	0.69
**HCC241-input**	HCC	19915649	13060838	0.66
**HCC290-input**	HCC	14729600	9148988	0.62
**HCC628-input**	HCC	13985304	9264263	0.66
**HCC324-input**	HCC	10706376	7158113	0.67
**pancreatic27-input**	pancreatic cancer	21005431	15092249	0.72
**pancreatic68-input**	pancreatic cancer	21609699	16086862	0.74
**pancreatic69-input**	pancreatic cancer	20381405	15078736	0.74
**pancreatic75-input**	pancreatic cancer	20150809	15023706	0.75
**pancreatic9-input**	pancreatic cancer	17788884	12676327	0.71
**pancreatic15-input**	pancreatic cancer	66823239	44834027	0.67
**pancreatic22-input**	pancreatic cancer	20343874	13994980	0.69
**GBM57-input**	GBM	16663028	12288601	0.74
**GBM58-input**	GBM	19745555	15066250	0.76
**GBM66-input**	GBM	22743166	16710159	0.73
**GBM76-input**	GBM	19426157	14503092	0.75
**stomach1-input**	gastric cancer	15593466	11708043	0.75
**stomach2-input**	gastric cancer	19726402	14537170	0.74
**stomach4-input**	gastric cancer	12241169	9077833	0.74
**stomach8-input**	gastric cancer	15604495	11004207	0.71

**colon13-input**	colorectal cancer	19419793	14360900	0.74
**colon16-input**	colorectal cancer	17016615	12384120	0.73
**colon17-input**	colorectal cancer	18873289	13685934	0.73
**colon19-input**	colorectal cancer	20046893	14638576	0.73
**BR7-input**	breast cancer	17555208	12726778	0.72
**BR13-input**	breast cancer	18015338	13467760	0.75
**HBV397-input**	HBV	12448351	8786039	0.71
**HBV640-input**	HBV	16143446	10547979	0.65
