# Supplementary material for: 5-Hydroxymethylcytosine signatures in cell-free DNA provide information about tumor types and stages
Source: Cell Res. 2017 Aug 18;27(10):1231–42. doi: 10.1038/cr.2017.106 (PMC5630676; doi:10.1038/cr.2017.106)
Supplement: Supplementary information, Table S11 — Top gene body feature set used for cancer prediction. [file cr2017106x21.pdf]

Table S11 Top gene body feature set used for cancer prediction.

CLDN15	SLC25A47	ZRANB2	LOC100506963	STXBP3	GPR26
P2RX2	LOC100507410	LHX5	HOXC5	FAM96A	CALCB
RNF223	SHISA2	SLAMF7	PAX1	DACH1	LOC100128946
ASF1B	KIF16B	SSR2	LARS	DHRS3	CCDC33
GMCL1P1	COMMD6	SPATA31E1	ABRACL	SAMD11	UBQLN4
TCEA3	SYT2	INSL4	RAG1	CCNL2	CRP
DDX11L1	LOC729737	WASH7P	LOC100132287		
