# Supplementary material for: 5-Hydroxymethylcytosine signatures in cell-free DNA provide information about tumor types and stages
Source: Cell Res. 2017 Aug 18;27(10):1231–42. doi: 10.1038/cr.2017.106 (PMC5630676; doi:10.1038/cr.2017.106)
Supplement: Supplementary information, Table S12 — Top DhMR feature set used for cancer prediction. [file cr2017106x22.pdf]

Table S12 Top DhMR feature set used for cancer prediction.

chr4:90790001-90792000	chr6:45304001-45306000	chr1:169422001-169424000
chr1:67584001-67586000	chr5:103492001-103494000	chr3:87312001-87314000
chr2:219148001-219150000	chr1:198222001-198224000	chr8:53686001-53688000
chr1:239846001-239848000	chr3:23318001-23320000	chr6:122406001-122408000
chr9:3496001-3498000	chr1:24806001-24808000	chr8:69672001-69674000
chr2:49900001-49902000	chr3:107894001-107896000	chr8:42934001-42936000
chr3:17352001-17354000	chr6:157286001-157288000	chr3:108506001-108508000
chr4:39342001-39344000	chr6:129198001-129200000	chr3:137070001-137072000
chr1:59248001-59250000	chr5:83076001-83078000	chr3:93728001-93730000
chr2:213134001-213136000	chr5:39530001-39532000	chr1:3234001-3236000
chr1:37824001-37826000	chr6:156800001-156802000	chr7:13364001-13366000
chr1:77664001-77666000	chr2:154460001-154462000	chr2:41780001-41782000
